# Urban and Rural Disparities in Hospital Utilization among Indonesian Adults

**Published:** 2019-02

**Authors:** Agung Dwi LAKSONO, Ratna Dwi WULANDARI, Oedojo SOEDIRHAM

**Affiliations:** 1. National Institute of Health Research and Development, Indonesian Ministry of Health, Jl. Percetakan Negara 29, Jakarta, Indonesia; 2. Doctoral Program, Faculty of Public Health, Universitas Airlangga, Surabaya, Indonesia; 3. Faculty of Public Health, Universitas Airlangga, Surabaya, Indonesia

**Keywords:** Disparities, Hospital utilization, Urban-rural, Indonesia

## Abstract

**Background:**

Equal access to healthcare facilities, patient’s satisfaction, and respect for the desire of the patient were recognized as the basic principles of each of the health care system. Each person must be given the opportunity to access health services in accordance with the requirements of their health. We aimed to prove the existence of disparities hospital utilization based on the category of urban-rural areas.

**Methods:**

The research used the 2013 Indonesian Basic Health Survey (RKD) as analysis material, that was designed a cross-sectional survey. With the multi-stage cluster random sampling method, 722,329 respondents were obtained. Data were analyzed using Multinomial Logistic Regression tests.

**Results:**

The results showed adults living in urban were likely to use hospital outpatient facilities 1.246 times higher than adults living in rural areas (OR 1.246; 95% CI 1.026 – 1.030). The likelihood of utilizing at the same time outpatient and inpatient facilities at 1.134 times higher in adults living in urban than those in rural areas (OR 1.134; 95% CI 1.025 – 1.255). While for the category of hospital inpatient utilization, there was no significant difference.

**Conclusion:**

There was a disparity in hospital utilization between urban-rural areas. Urban show better utilization than rural areas in outpatient and at the same time the use of inpatient care.

## Introduction

High disparities between regions are often caused by centralized economic activities and development in one particular region. Often the centrality of economic activities occurs in urban areas. The end result is disparities that occur in all fields, no exception in the field of health. The hospital as a health service facility reference from the basic level is often built in urban. The policy to build a hospital in this urban area can be understood. The major reason is to ensure community access easier because of the availability of a better means of transportation, both on public transportation and infrastructure (1).

Equal access to hospital, satisfaction patients, and respect for the desire of the patient has been recognized as a basic principle of every health services system (2). Unlinking or devalue disparities utilization of health services is the concentration of health planners and policy makers (3). This must be done as one of the efforts to improve the health care system performance indicator.

Indonesia has the geographic problem as a natural barrier to provide fair health services for the community. From 17,504 islands which belong to Indonesia, at least around 16,056 islands have been verified by the United Nations Group of Experts on Geographical Names (UNGEGN)(4). Other natural barrier is the varied tribe of the people in Indonesia who also have their own local language. There are at least 1,300 tribes (5). This condition increases the challenges must be faced in ensuring equal access.

The presence of barriers does not eliminate the government’s obligation to guarantee equal access to health services. We aimed to prove the existence of disparities hospital utilization based on the category of urban-rural areas.

## Materials and Methods

Hospital utilization data that used in the analysis of this research were the results of the 2013 RKD data. The utilization of the hospital covers the hospital owned by the government and the private sector. The unit of analysis in this research was the Indonesian population fifteen years old and above. At that age, the respondent was assumed to be an adult, could make his own decision to utilize the hospital or not. This reason was taken because information on hospital utilization was based on respondents’ acknowledgment. RKD has done with sample 1,027,763 individuals. The sample analysed was based on the analysis unit Indonesian adult of 722,329 respondents (6).

The utilization of the hospital was the community access to the hospital, either does outpatient or inpatient. Inpatient was the respondent’s acknowledgment of the use of hospitalization in the hospital last year. Outpatient was the respondent’s acknowledgment of the use of outpatient care at the hospital for the past month. Insurance type was the respondents’ acknowledgment of insurance ownership which was divided into 3 categories, namely having no insurance, government-run insurance (Askes, Jamkesmas, Jamkesda, Jamsostek), and private-run insurance. Socioeconomic status was the index of goods ownership quintile stated by the respondent (6).

Data were obtained through a structured questionnaire. The variables analyzed included age, gender, marital status, education level, work type, socioeconomic status, insurance, travel time and transportation cost. Statistical analysis done started using Chi-Square for dichotomy variables and t-tests for the continuous variable.

This test was used to assess whether there are differences in urban and rural significant statistically. Because of the nature of the dependent variables, estimation using Multinomial Logistic Regression. All analytics by SPSS 19 software (Chicago, IL, USA).

### Ethical approval

The 2013 RKD survey has an ethical clearance that was approved by the national ethical committee (ethic number: 01.1206.207). Informed consent was used during data collection, which was considered aspects of data collection procedures, voluntary, and confidentiality.

## Results

Before conducting a multinomial logistic regression test, a co-linearity test was carried out. Table 1 shows the results of co-linearity tests show that there is no co-linearity between dependent variables and independent variables.

**Table 1:** Results for co-linearity test

***Variables***	***Sig.***	***Collinearity Statistics***	
		***Tolerance***	***VIF***
Urban/rural	0.000	0.700	1.429
Age	0.000	0.541	1.847
Gender	0.042	0.831	1.204
Marital status	0.000	0.537	1.861
Education level	0.000	0.714	1.401
Work type	0.000	0.806	1.241
Socioeconomic status	0.000	0.671	1.490
Insurance	0.000	0.987	1.014
Travel time	0.000	0.572	1.749
Transportation cost	0.000	0.594	1.684

* Dependent Variable: Hospital utilization

### Descriptive Results

Table 2 shows that there is a difference between the adult in rural and urban areas for all the observed characteristics are statistically significant. Table 2 shows that the average of the people who live in urban slightly younger than in the rural area. Indonesia adult dominated by women than men, also dominant with married status and education levels below the elementary school.

**Table 2:** Descriptive statistic of hospital utilization among Indonesia adult

***CHARACTERISTIC***	***AREA***		***ALL***	***P***
	***URBAN***	***RURAL***		
Hospital Utility^*^				0,000
• Outpatient	5030 (1.5%)	2742 (0.7%)	7772 (1.1%)	
• Inpatient	6753 (2.0%)	5029 (1.3%)	11782 (1.6%)	
• Outpatient + inpatient	1444 (0.4%)	832 (0.2%)	2276 (0.3%)	
• No utilization	320504 (96.0%)	379995 (97.8%)	700499 (97.0%)	
Age (mean)^**^	333731 (39.62)	388598 (41.8)	722329 (39.92)	0.000
Gender^*^				0.000
• Male	159227 (47.7%)	188596 (48.5%)	347823 (48.2%)	
• Female (Ref.)	174504 (52.3%)	200002 (51.5%)	374506 (51.8%)	
Marital status ^*^				0.000
• Single	84459 (25.3%)	82276 (21.2%)	166735 (23.1%)	
• Married	222530 (66.7%)	276232 (71.1%)	498762 (69.0%)	
• Divorce (Ref.)	26742 (8.0%)	30090 (7.7%)	56832 (7.9%)	
Education level ^*^				0.000
• Primary school and under	115974 (34.8%)	232779 (59.9%)	348753 (48.3%)	
• Junior high school	70479 (21.1%)	77177 (19.9%)	147656 (20.4%)	
• Senior high school	110861 (33.2%)	64488 (16.6%)	175349 (24.3%)	
• College (Ref.)	36417 (10.9%)	14154 (3.6%)	50571 (7.0%)	
Work type ^*^				0.000
• No work	146466 (43.9%)	145513 (37.4%)	291979 (40.4%)	
• Public servant/army/police	21648 (6.5%)	10882 (2.8%)	32530 (4.5%)	
• Employee	35939 (10.8%)	14142 (3.6%)	50081 (6.9%)	
• Entrepreneur	59280 (17.8%)	33850 (8.7%)	93130 (12.9%)	
• Farmer/Fisherman/Labor	53957 (16.2%)	170687 (43.9%)	224644 (31.1%)	
• Others (Ref.)	16441 (4.9%)	13524 (3.5%)	29965 (4.1%)	
Socioeconomic ^*^				0.000
• Quintile 1	16592 (5.0%)	116155 (29.9%)	132747 (18.4%)	
• Quintile 2	40704 (12.2%)	98949 (25.5%)	139653 (19.3%)	
• Quintile 3	70242 (21.0%)	76532 (19.7%)	146774 (20.3%)	
• Quintile 4	96423 (28.9%)	54969 (14.1%)	151392 (21.0%)	
• Quintile 5 (Ref.)	109770 (32.9%)	41993 (10.8%)	151763 (21.0%)	
Insurance ^*^				0.000
• No insurance	146160 (43.8%)	166386 (42.8%)	312546 (43.3%)	
• Government-run insurance	174916 (52.4%)	218661 (56.3%)	393577 (54.5%)	
• Private-run insurance (Ref.)	12655 (3.8%)	3551 (0.9%)	16206 (2.2%)	
Travel time ^*^				0.000
• ≤ 30 Minutes	203688 (61.0%)	76641 (19.7%)	280329 (38.8%)	
• > 30 Minutes (Ref.)	130043 (39.0%)	311957 (80.3%)	442000 (61.2%)	
Transportation Cost ^*^				0.000
• ≤ IDR 15.000	239340 (71.7%)	126871 (32.6%)	366211 (50.7%)	
• > IDR 15.000	94391 (28.3%)	261727 (67.4%)	356118 (49.3%)	

* Chi-Square test was used for dichotomous variables./

** T-test for continuous variables.

Table 2 also shows that the adults who live in urban dominated by those who do not have work, while in the rural area is dominated by adults who worked as farmers/labor/fishermen. Socio-economic characteristics of adults who live in urban dominated by quintile 5 (very rich), while in rural dominated by adults who include in quintile 1. The characteristics of ownership of insurance are dominated by adults who have insurance in whole area.

The information in Table 2 also shows that time travel to the hospital in the urban area is dominated by the shortest less than 30 minutes, while in the rural area is dominated by the time travel more than 30 minutes. This condition in relation to the transportation cost required to reach the hospital. In urban areas is dominated by transportation costs less than IDR 30.000, while in the rural area is dominated by more than IDR 30.000.

The striking difference between adults who use hospitals in urban-rural areas is those who utilize at the same time outpatient and inpatient care. Adults who live in urban areas use twice as much as those who live in rural areas. As for outpatients in hospitals, adults living in urban areas are also almost twice as many as adults living in rural areas. While for inpatients though more in adults who live in urban areas, but not too far away.

**Fig. 1:**
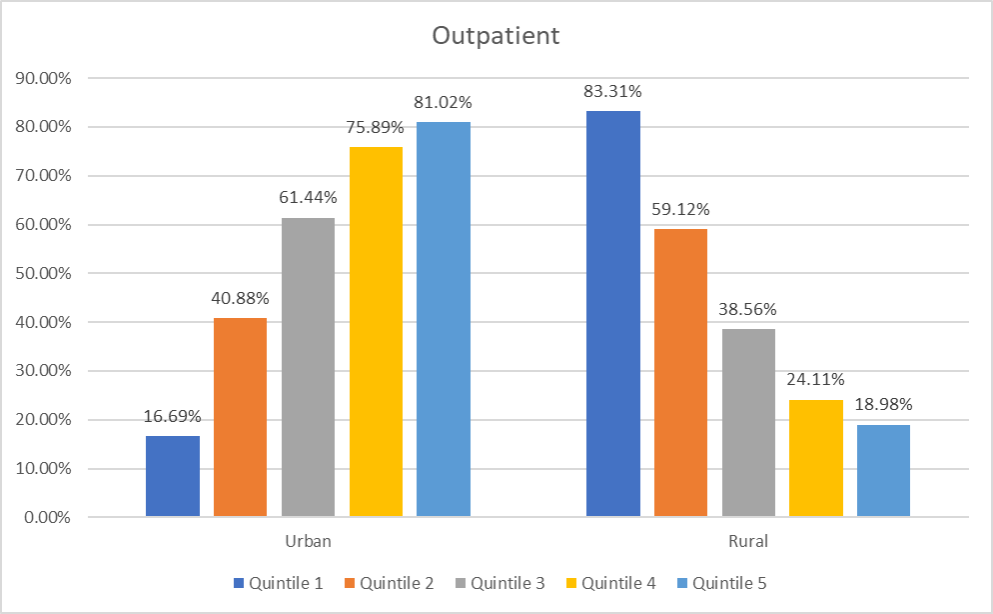
Distribution of hospital outpatient utilization in Indonesian adults

**Fig. 2:**
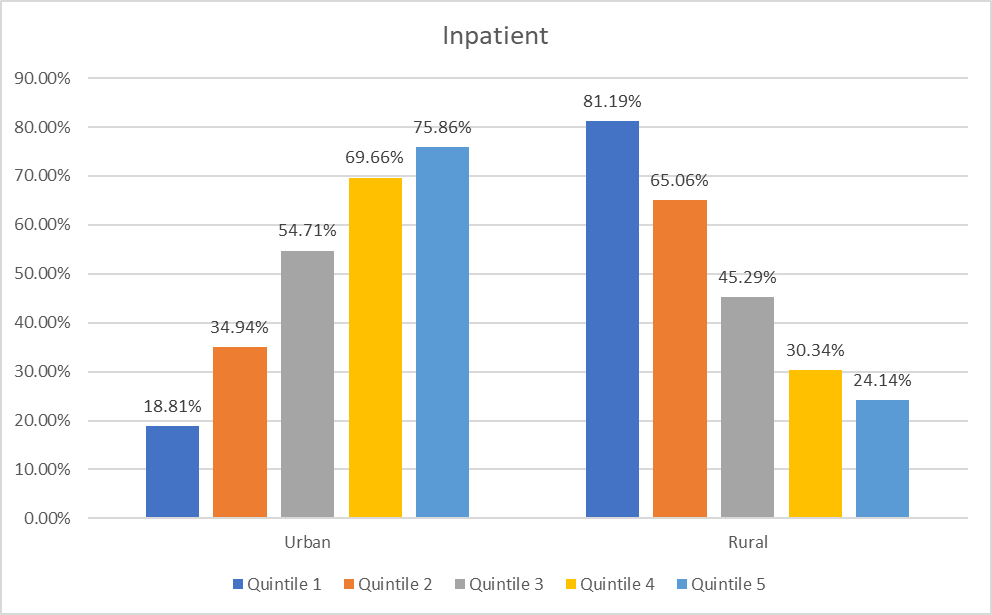
Distribution of hospital inpatient utilization in Indonesian adults

**Fig. 3:**
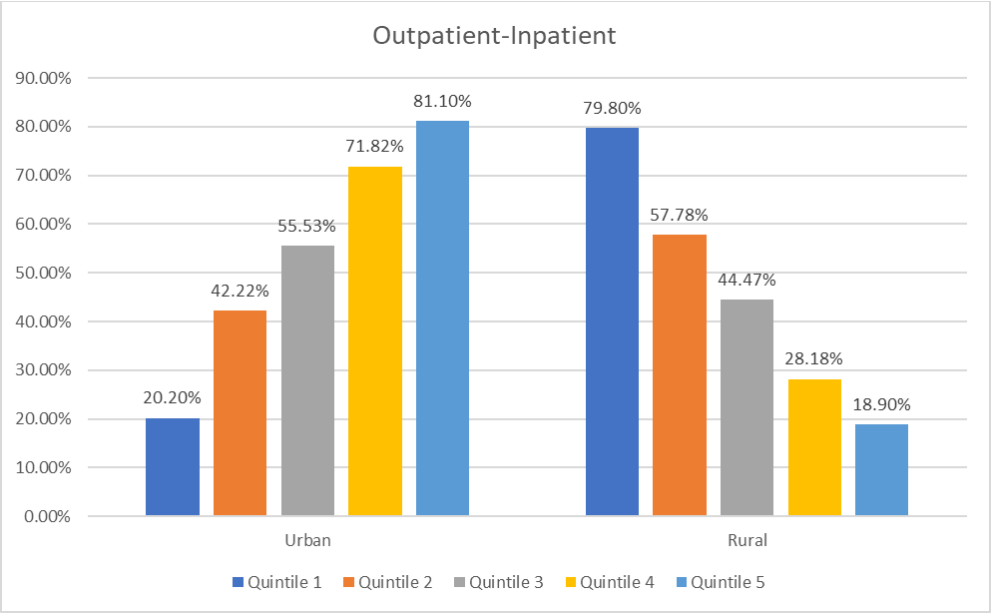
Distribution of hospital outpatient-inpatient utilization in Indonesian adults

Figs. 1–3 show that in urban areas, rich people (quintile 5) are the most utilizing hospital services. The opposite condition applies in rural areas, poor people (quintile 1) are the most utilizing hospitals. This situation applies to all hospital services.

### Multivariate Regression Analyses

Table 3 displays the results of multinomial logistic regression tests to illustrate the difference between the utilization of the hospital in urban and rural areas. As a reference selected category is “no utilization”. Table 3 shows a clear disparity between the adults in the urban and rural area who use the outpatient services at the hospital. Those who live in urban areas may utilize outpatient facility hospital 1.246 times higher than adults who live in rural areas (OR 1.246; 95% CI 1.026–1.030). The possibility of utilizing at the same time outpatient and inpatient facilities at 1.134 times is higher in adults living in urban areas than those in rural areas (OR 1.134; 95% CI 1.025–1.255). While for the category of hospital inpatient utilization, there is no significant difference.

**Table 3:** Multinomial logistic regression of hospital utilization among Indonesia adult

***Predictor***	***Outpatient***			***Inpatient***			***Outpatient + Inpatient***		
	***OR***	***Lower Bound***	***Upper Bound***	***OR***	***Lower Bound***	***Upper Bound***	***OR***	***Lower Bound***	***Upper Bound***
Age	1.028^*^	1.026	1.030	1.015^*^	1.013	1.017	1.032^*^	1.029	1.036
Area: Urban	1.246^*^	1.178	1.318	1.043	0.999	1.090	1.134^*^	1.025	1.255
Gender: Male	0.981	0.932	1.032	0.936^*^	0.897	0.976	1.220^*^	1.109	1.342
Marital Status: single	0.990	0.879	1.115	0.767^*^	0.697	0.844	0.890	0.710	1.116
Marital Status: married	1.120^*^	1.031	1.215	1.033	0.966	1.104	1.250	1.079	1.447
Edu: primary school & under	0.562^*^	0.512	0.616	0.798^*^	0.736	0.865	0.693^*^	0.583	0.824
Edu: junior high school	0.734^*^	0.669	0.807	0.821^*^	0.755	0.892	0.893	0.748	1.067
Edu: senior high school	0.838^*^	0.774	0.907	0.854^*^	0.793	0.921	0.907	0.775	1.060
Work: No work	1.275^*^	1.134	1.433	1.204^*^	1.099	1.320	1.467^*^	1.190	1.809
Work: Public servant/ army/police	1.166^*^	1.015	1.340	0.909	0.806	1.025	0.830	0.638	1.080
Work: Employee	1.145^*^	1.001	1.310	0.940	0.841	1.050	0.852	0.661	1.098
Work: Entrepreneur	0.845^*^	0.742	0.962	0.826^*^	0.746	0.915	0.749^*^	0.592	0.947
Work: Farmer/fisherman/labor	0.789^*^	0.696	0.895	0.745^*^	0.675	0.821	0.640^*^	0.509	0.804
Socioeconomic: quintile 1	0.723^*^	0.657	0.794	0.643^*^	0.596	0.695	0.545^*^	0.454	0.653
Socioeconomic: quintile 2	0.672^*^	0.618	0.731	0.818^*^	0.767	0.873	0.682^*^	0.587	0.791
Socioeconomic: quintile 3	0.789^*^	0.735	0.848	0.909^*^	0.858	0.963	0.755^*^	0.662	0.860
Socioeconomic: quintile 4	0.914^*^	0.859	0.971	1.004	0.953	1.058	0.929	0.831	1.039
Insurance: No insurance	0.274^*^	0.247	0.303	0.455^*^	0.413	0.502	0.238^*^	0.196	0.288
Insurance: Government-run insurance	0.569^*^	0.518	0.626	0.708^*^	0.644	0.779	0.555^*^	0.465	0.662
Travel time: ≤ 30 Menit	1.419^*^	1.337	1.505	1.263^*^	1.205	1.325	1.259^*^	1.132	1.401
Transport cost: ≤IDR 15.000	1.238^*^	1.165	1.316	1.359^*^	1.295	1.427	1.517^*^	1.356	1.698

* Significant at 95% level

Table 3 also shows the disparities seen in other categories. Adults who have better education tend to be more utilizing hospitals in all categories, both outpatient, inpatient, as well as the combination of both services. This condition is directly proportional to socioeconomic status, the better the socioeconomic status of adults in Indonesia, the more likely it is to use hospital services in all categories. The insurance ownership category shows that those who have government-run insurance have better hospital utilization than those who do not have insurance. While those who have insurance managed by the private sector are better at utilizing their hospital services than those who have insurance managed by the government. This condition applies to all categories of hospital utilization.

The information in Table 3 also shows that disparity in hospital utilization also occurs in the travel time and transportation costs categories. The possibility of greater use in adults in Indonesia has a faster travel time and cheaper transportation costs to hospitals.

## Discussion

As in most developing countries, the development of urban areas in Indonesia is more advanced than development in rural areas. This makes urban areas a special attraction for job seekers. The invasion of job seekers from rural to urban areas resulted in the proportion of unemployed in the urban population is higher than in rural. Majority of rural communities have very low levels of education, namely under the primary school, making the socioeconomics of rural communities not too good. Most rural people are in quintile 1. This condition is the opposite of the socioeconomic picture of the city.

Socioeconomic status has a close relationship with the patterns of disease, and indirectly to people’s access to hospitals. This socioeconomic aspect is indirectly explained in a study in Iran about the dietary and physical activity habits. The researchers found that those who had a low socioeconomic level tended to have better physical activity, while those who were rich had a better dietary pattern. The different dietary and physical activity habits in different socioeconomic status will make different demands on the hospital (7).

The results show that there are disparities in hospital utilization between urban-rural areas in Indonesia. WHO states that gender, education, occupation, income, ethnicity, and place of residence are factors that influence the accessibility of health services (8). The results of this study again prove that almost all of these factors exist as predictors of in hospital utilization disparities in Indonesia. Disparities of health services utilization related to rurality do not only happen in Indonesia. The research results with the focus of disparities in many countries reporting the disparities existing, among others in China (3)(9), Canada (10), The USA (11)(12), Ethiopia (13)(14), Mongolia (15), Australia (1), and Taiwan (16)(17). In addition to urban and rural, disparities in many countries also reportedly happened on many categories. Among them is the socio-economic status (9)(13)(15)(18)(19)(20)(21)(22), the status of ownership of insurance (3)(9)(23), the status of the level of education (13), Ethnic (24)(25)(26) and geographic (27)(28).

The findings in this study illustrate that there are still obstacles to access to health services by rural communities. Access to health services is an important indicator that illustrates the fulfillment of quality health care needs for the community (29), has not been well fulfilled in rural communities in Indonesia. This access barriers mainly occur due to long travel time (>30 minutes), and transportation cost more expensive (> IDR 15,000).

For the rural community which has dominant in poor. Travel time and transportation cost become important variables are taken into consideration. The long travel time means increasing the opportunity lost for those who work, and expensive transportation costs mean a big sacrifice. This is in line with a study in Asian countries which mentions that the factors that affect the utilization of hospital in addition to the costs of service delivery is the other costs such as transport, patient food, accommodation and opportunity costs (30).

Assessment of the sacrifice costs, will affect the results will affect the decision to use or not use the hospital. If the sacrifice is greater than the benefits obtained, then the decision taken is usually a delay of the hospital utilization. If this is allowed to happen in the long term, one of the securities that will arise is the increasing number of chronic diseases. A study in Northeast China found that the prevalence of chronic diseases such as hypertension, chronic ischemic heart disease, cerebrovascular disease, chronic low back pain, arthritis chronic, suffering/peptic ulcer, higher on rural communities (31). Research in Hubei Province-China also found the same. The higher life expectancy of the inhabitants of rural but not accompanied by easier access makes the rural population more potentially experiencing chronic disease (32).

These findings indicate that reduce hospital utilization disparities between urban-rural is the exact steps to improve the community health status. Specific efforts must be taken so that various barrier the hospital utilization can be eliminated gradually. Improvement efforts through the implementation of national health insurance in Taiwan that has been running in fifteen years also have not been able to eliminate disparities hospital utilization between urban-rural (16)(17). The effort to reduce rural-urban disparities in hospital utilization require focused on the general issues in rural areas such as poverty, living conditions, lack of education, and lack of health information (31).

In a different context in Iran, several studies were conducted to detect disparities between regions. A research (33) found regional disparities in Iranian society in accessibility to healthcare resources. There was a relationship between the regional disparity of obstetrics and gynecologic services with children and infants mortality rates in Iran (34), while regional disparities were proven to exist in the distribution of health care facilities in Iran (35). Spatially Rostami et al (36) also proved the existence of geographic disparity in fatal drug overdose cases, while Mansori et al (37) and Momenyan et al (38) found a spatial disparity. Mansori et al (37) detected disparity in the incidence of colorectal cancer case, and Momenyan et al (38) in HIV/AIDS cases mortality risk.

## Conclusion

Based on the description of the research results, it can be concluded that there is a disparity between urban and rural areas in the hospital utilization as outpatient and outpatient-inpatient at the same time in Indonesia. The disparity in hospital utilization is also found in other categories, namely gender, marital status, education level, work type, socioeconomic, insurance, travel time and transportation cost. To reduce or minimize the disparity in the hospital’s utilization, policymakers can make the results of this study as a guide to choosing the focus of the service equity policy.

## Ethical considerations

Ethical issues (Including plagiarism, informed consent, misconduct, data fabrication and/or falsification, double publication and/or submission, redundancy, etc.) have been completely observed by the authors.
